# Modifying strategies for SDF-1/CXCR4 interaction during mesenchymal stem cell transplantation

**DOI:** 10.1007/s11748-021-01696-0

**Published:** 2021-09-12

**Authors:** Qin Jiang, Keli Huang, Fang Lu, Shaoping Deng, Zhenglin Yang, Shengshou Hu

**Affiliations:** 1 Department of Cardiac Surgery, Sichuan Provincial People’s Hospital, Affiliated Hospital of University of Electronic Science and Technology, Chengdu, China; 2 Medico-Engineering Cooperation On Applied Medicine Research Center, University of Electronic Science and Technology of China, Chengdu, China; 3 Department for Organ Transplantation, Sichuan Academy of Medical Science, Sichuan Provincial People’s Hospital, Chengdu, Sichuan China; 4 The Key Laboratory for Human Disease Gene Study of Sichuan Province and the Department of Laboratory Medicine, Sichuan Provincial People’s Hospital, University of Electronic Science and Technology of China, No. 32, West Second Section First Ring Road, Chengdu, 610072 Sichuan China; 5 Department of Cardiac Surgery, Fuwai Hospital, National Center for Cardiovascular Disease, Chinese Academy of Medical Sciences and Peking Union Medical College, Beijing, China

**Keywords:** Mesenchymal stem cells, Cell transplantation, Myocardial infarction, Stromal cell-derived factor-1, CXCR4 receptor

## Abstract

Mesenchymal stem cell (MSC) transplantation is regarded as a promising candidate for the treatment of ischaemic heart disease. The major hurdles for successful clinical translation of MSC therapy are poor survival, retention, and engraftment in the infarcted heart. Stromal cell-derived factor-1/chemokine receptor 4 (SDF-1/CXCR4) constitutes one of the most efficient chemokine/chemokine receptor pairs regarding cell homing. In this review, we mainly focused on previous studies on how to regulate the SDF-1/CXCR4 interaction through various priming strategies to maximize the efficacy of mesenchymal stem cell transplantation on ischaemic hearts or to facilitate the required effects. The strengthened measures for enhancing the therapeutic efficacy of the SDF-1/CXCR4 interaction for mesenchymal stem cell transplantation included the combination of chemokines and cytokines, hormones and drugs, biomaterials, gene engineering, and hypoxia. The priming strategies on recipients for stem cell transplantation included ischaemic conditioning and device techniques.

Mesenchymal stem cells (MSCs) are derived from multiple biological tissues, such as adult bone marrow, adipose tissues, and neonatal tissues, such as the umbilical cord and placenta [1]. MSCs are promising for therapeutic applications given the ease of obtaining them, their genetic stability, their poor immunogenicity, and their curative properties for tissue repair and immunomodulation [2]. A number of clinical trials using MSCs for therapeutic interventions have been carried out for cardiovascular diseases and severe degenerative and/or inflammatory diseases, alone or in combination with other drugs [3]. Although the mechanisms by which stem cells take effect in injured tissue remain debated [4], a modest but consistent improvement in cardiac function in animal models has been obtained [5]. However, MSCs encounter hostile environments due to oxidative stress, severe hypoxia, and nutrient deficiency, and are thus more likely to undergo apoptosis during the early days posttransplantation. This situation is further exacerbated by poor clearance of metabolic waste due to loss of functional vascular networks at the injury site [6]. Various other problems, such as low transplanted cell viability, poor homing and engraftment into injured tissues, MSC heterogeneity, and lack of adequate information on optimum MSC doses, impede clinical applications [7]. In fact, the transplanted efficacy of MSC retention on the heart after intravenous delivery (1%) was extremely low even at the maximum period of acute myocardial infarction (AMI) [8]. Systemic redistribution results in a large number of transplanted MSCs being frustrated and trapped in lung capillaries, preventing them from accessing target tissues [9]. MSCs express procoagulant activity due to the surface expression of tissue factor, which initiates coagulation when entering vessels. Simultaneous activation of both the coagulation and complement pathways is called an instant blood-mediated inflammatory reaction, which could also result in low engraftment rates and discouraging results in clinical trials [10].

The MSC-sourced secretome, defined as a series of MSC-derived bioactive factors (soluble proteins, nucleic acids, lipids, and extracellular vesicles), showed therapeutic effects similar to those of MSC transplantation. The MSC-derived secretome has an advantage over cells, because it bypasses many side effects of MSC-based therapy, including unwanted differentiation of engrafted MSCs [11]. However, there are numerous limitations linked to the use of extracellular vesicles (EVs) as therapeutic vehicles, including the difficulty of efficiently loading therapeutics into EVs, preventing clearance of the EVs from circulation, engrafting on the target tissue, and the insufficiency of internalization and functional transfer of the cargo [12].

It has been extensively demonstrated that MSC homing and engraftment are regulated by the interaction between stromal-derived factor 1 (SDF-1) and C-X-C motif receptor 4 (CXCR4). In addition, some studies have confirmed that the expression level of SDF-1 is significantly increased after cardiac ischaemia, and the recruitment of MSCs expressing CXCR4 towards the SDF-1 gradient plays a crucial role in tissue recovery [13]. On the other hand, implantation of MSCs into the ischaemic myocardium could improve the expression levels of collagen protein, SDF-1, and vascular endothelial growth factor (VEGF) in the infarcted borderline region [14]. SDF-1 and CXCR4 are significantly upregulated in the heart in both animal and human studies of AMI [15]. The cardiac SDF-1α–CXCR4 interaction is also present and capable of providing protection against ischaemia/reperfusion-induced injury [16]. However, the increase in SDF-1 expression peaked at 1 week after AMI and gradually decreased later. The dynamic change is well documented by nucleic acid and protein detection technology [17] and reflected in ultrasound molecular imaging [18]. SDF-1 delivery to the myocardium after AMI is associated with improvements in stem cell homing, angiogenesis, and left-ventricular function in animal models and enhancements in heart function and quality of life in humans. Despite these potential pleiotropic effects, the SDF-1 protein is limited by its short plasma half-life due to the cleavage effect of dipeptidylpeptidase-4 (DPP-4) [19]. Moreover, SDF-1 did not significantly improve the proportion of circulating cells that adopted cardiomyocyte fates [20].

Therefore, strengthening the important interaction by regulating SDF-1/CXCR4 expression in the ischaemic heart during MSC transplantation is a potential solution to the limited transplantation efficacy. To overcome this challenge, scientists have established several strategies to generate highly functional MSCs or/and friendly homing environments [21]. In aggregate, based on the experimental observations that the effective homing of the exogenously transplanted cells greatly improves the efficacy of cells to integrate and function in the target tissues, this review discusses the successful treatments for how to modify SDF-1/CXCR4 interaction in two main aspects: the factors that increase the ability of stem cells to respond to the migratory stimuli, including pre-conditioning of MSCs with various stimulants such as inflammatory agents and medication, genetic manipulation and modification of culture conditions with hypoxic culture (Fig. 1); and the methods for modulating the target sites to be more attractive for stem cell recruitment, including ischaemic conditioning and device technique (Fig. 2) [22].

**Fig. 1:**
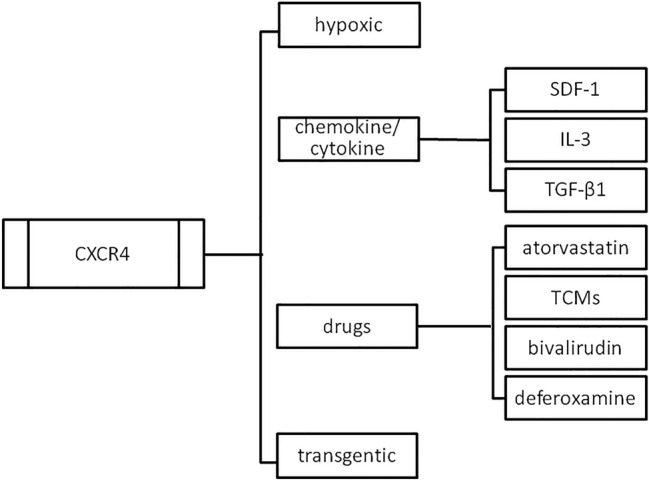
The strategies to strengthen CXCR4 during MSC transplantation for cardiac repair to increase the ability of stem cells to respond to the migratory stimuli, including pre-conditioning of MSCs with various stimulants such as chemokines agents, and medication, genetic manipulation, and modification of culture conditions with hypoxic culture

**Fig. 2:**
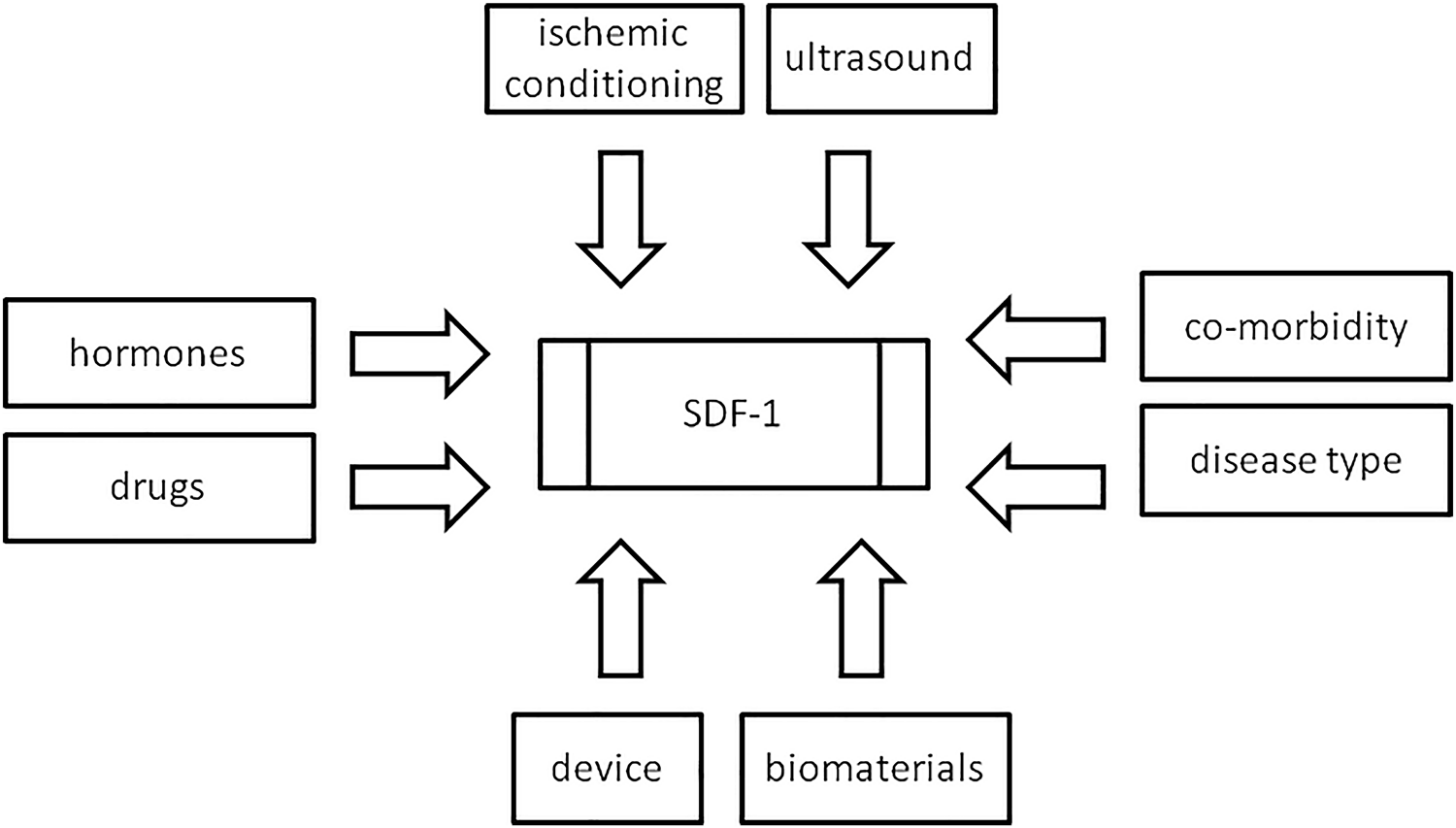
The strategies to strengthen SDF-1 during MSC transplantation for cardiac repair with modulation of the target sites to be more attractive for stem cell recruitment, including hormones, drug, ischemic conditioning, ultrasound, device technique, biomaterials, co-morbidity, and disease conditions

## Chemokines and cytokines

Chemokine preconditioning is a quick and simple way to enhance stem cell survival and regeneration of an infarcted myocardium. Preconditioning with the chemokine SDF-1 suppresses MSC apoptosis, enhances MSC survival, engraftment, and vascular density, and improves myocardial function by augmenting SDF/CXCR4 signalling [23]. Interleukin-3 (IL-3) preconditioning upregulates the expression of CXCR4 on MSCs, which leads to increased cell migration towards the gradient of SDF-1α [24]. TGF-β1 induces increased CXCR4 expression and MSC homing in the repair process of rat myocardial injury by regulating the expression of CXCR4 on the cell membranes, and the inhibitory effect of the anti-CXCR4 antibody was more evident than that of the anti-TGF-β1 antibody in TGF-β1 treatment in an in vivo homing experiment, which indicates that the SDF/CXCR4 interaction played a pivotal role [25]. Similarly, pre-exposure of human MSCs to TGF-β results in enhanced homing ability through increasing CXCR4 expression; diminishing homing capacity has been evidenced when the inhibitor of the TGF-β receptor and CXCR4 receptor is applied [26].

## Medications

As a low-molecular-weight protein, SDF-1 can be degraded by proteases, including matrix DPP-4/CD26, which exists in the circulatory system and is activated in ischaemic tissues. It appears to maintain the optimal endogenous SDF-1 concentration by inhibiting degradation proteases and then enhancing the homing capacity of progenitor cells [27]. DPP4 cleaves and inactivates a wide variety of substrates, including SDF-1. DPP4 truncates enzymatically within the N-terminal amino acid of granulocyte colony-stimulating factors (G-CSF), granulocyte–macrophage colony-stimulating factors (GM-CSF), erythropoietin, and human IL-3, and decreases their activity; such truncation is blocked by diprotin A [28].

In the course of experiments showing that inhibition of DPP4 potentiates SDF-1-mediated progenitor cell survival, the potential clinical utility of DPP4 inhibition by other similar molecular structures enhanced engraftment in the ischaemic myocardium by reducing SDF-1 degradation. Stem cell mobilization by G-CSF and stabilization of cardiac SDF-1 by genetic or pharmacological DPP4 inhibition (i.e., the therapeutic concept of dual strengthening strategies on stem cell therapy) was shown to enhance heart function and survival after MI in mice [29]. In vitro and in vivo, parathyroid hormone inhibited the activity of DPP4, which reduced SDF-1 cleavage and inactivation. Parathyroid hormone-treated mice also showed significantly improved homing of CXCR4-positive stem cells associated with an increased protein level of the corresponding homing factor SDF-1 in the ischaemic heart [30]. Erythropoietin-treated animals had remarkably enhanced survival post-MI. The subpopulations (CD31 + , c-kit + , and Sca-1 + cells) were mobilized, and homing of Sca-1 + and CXCR4 + stem cells towards an SDF-1 gradient into the ischaemic myocardium was enhanced [31]. SDF-1 expression is compromised after AMI in diabetic mice, but overexpression of the SDF-1 gene could mitigate the adverse effect [32]. Sitagliptin, as a DPP-4 inhibitor, is an antihyperglycaemic agent that prevents N-terminal cleavage and inactivation of the incretin hormones glucagon-like peptide-1 and glucose-dependent insulinotropic peptide. It does not affect tubuloglomerular feedback or impair renal haemodynamic function, and findings relevant to using DPP4 inhibitors for treating type 2 diabetes mellitus are present with increased levels of intact SDF-1α1-67 [33].

Tadalafil treatment results in higher expression of SDF-1 in MSCs, prolongs MSC survival via upregulation of miR-21-dependent suppression of Fas, and increases stem cell mobilization and homing into the infarcted myocardium as a result of cardiac function improvement [34]. Atorvastatin-pretreated MSCs with an adjunct of atorvastatin intragastric administration significantly facilitate targeted recruitment to peri-infarct myocardium and result in further protection in cardiac apoptosis and inhibition of adverse ventricular remodelling after AMI via SDF-1/CXCR4 signalling [35].

Traditional Chinese medicines have been investigated for their ability to enhance stem cell homing in MI. Danhong injection, which is characteristic of ameliorating blood circulation, can increase the expression of CXCR4 and SDF-1 in MSCs and the myocardium, respectively. This effect can be prohibited by the SDF1/CXCR4 antagonist [36]. Guanxin Danshen formulation (the product mainly extracted from traditional Chinese medicine for ischaemic heart diseases) can further increase SDF-1 levels in the infarcted area and the number of injected MSCs in the infarct area compared with isolated cell transplantation [37]. Resveratrol (RSV), a plant polyphenol, has been identified in the root extract of white hellebore and *Polygonum cuspidatum*. RSV is a natural agent with powerful therapeutic potential for protecting against acute or chronic injury in multiple tissues due to its antioxidative, anti-inflammatory, and anticancer properties [38]. In accordance with its demonstrated properties, RSV may improve the therapeutic effects of MSCs by enhancing their engraftment, potentiating the paracrine mechanism, increasing lineage differentiation, and preventing senescence and ageing. In AMI model, RSV enhanced cardiac SDF-1 excretion via SIRT1 normalization/p53 inactivation pathway [39].

To prevent the procoagulant activity of MSCs, using anticoagulant drugs is of potential value. In contrast to heparin, which mechanistically blocks the SDF-1/CXCR4 axis by binding to the receptor and the ligand, bivalirudin, a thrombin inhibitor, has not been shown to prevent MSCs from homing and migrating in vitro and in vivo and might be a candidate for cell storage and for intracoronary MSC therapy in clinical practice [40]. Treatment of MSCs with deferoxamine, an iron chelator, increases the protein expression of HIF-1a and CXCR4, and MMP-2 and MMP-9 activity is significantly increased. The migration ability and homing activity of deferoxamine-conditioned MSCs are also significantly elevated in vitro and in vivo, respectively [41]. Diazoxide, a mitochondrial ATP-sensitive potassium channel opener, is effective in promoting MSC survival under oxidative stress and therapeutic efficacy in MI, but whether the mechanism is linked to SDF-1/CXCR4 signalling is still uncertain [42].

## Biomaterials

Biomaterials have been extensively investigated in terms of their ability to improve the therapeutic efficacy of MSC transplantation by providing suitable three-dimensional delivery carriers and sustaining cell viability and function [43]. The regeneration of chronically infarcted myocardium has been tested with injectable biomimetic hydrogels containing two therapeutic factors, SDF-1 and angiogenic peptides, which were used to strengthen stem cell homing and angiogenesis, respectively. The research results indicated that combined administration of SDF-1 and angiogenic peptides can result in more stem cell recruitment to the microenvironment, enhancement of angiogenic gene expression, increase neovascularization, and improvement of cardiac function in chronic MI [44]. Another cardiovascular active molecular nitric oxide (NO) release system has also been constructed with a hydrogel (NapFF-NO). The novel system was naphthalene covalently conjugated to a short peptide, FFGGG, and β-galactose caged nitric oxide (NO) donor, which can release NO molecules in response to β-galactosidase. The results showed that secretion of angiogenic factors VEGF and SDF-1 from MSCs was higher in the presence of NapFF-NO hydrogel, which can further enhance the therapeutic potential of MSCs for MI by increasing cell engraftment and paracrine action [45]. One novel SDF-1α transport device used coaxial electrospraying, which was incorporated into PLGA particles, presented a distinct core–shell structure reflected by transmission electron microscopy. The controlled delivery of SDF-1α from container PLGA and PLGA/bovine serum albumin particles maintained its chemotactic activity and increased the penetration capacity of MSCs into the injured myocardium [46]. The combination of CXCR-engineered MSC patches with diprotin A pretreatment inhibits myocardial ischaemia-induced injury, promotes tissue regeneration, and improves cell migration and recruitment, resulting in enhanced left-ventricular mechanical function after MI [47].

Using an artificial synthetic protein consisting of an SDF-1 domain and a glycoprotein VI (GPVI) domain, which have high binding affinity to CXCR4 and extracellular matrix proteins, respectively, has shown higher therapeutic efficacy in animal experiments. SDF-1–GPVI results in CXCR4-positive cell mobilization, enhances bone marrow cell survival and endothelial differentiation in vitro, and demonstrates proangiogenic effects in vivo. In a mouse MI model, administration of the structure domain-modified protein led to significant enhancement in terms of cell recruitment, capillary density, and cardiac function [48]. The “death cell-chasing” protein AnxA5 was fused into SDF-1 protein to trace the injured myocardium. The receptor competition assay demonstrated that SDF-1–AnxA5 had high binding affinity to the SDF-1 receptor CXCR4. SDF-1–AnxA5 administered by intravenous injection could accumulate at the infarcted myocardium in vivo. Treatment with SDF-1–AnxA5 ameliorated cardiac function because of its directional migration, which is characteristic of special binding to dead cells [49].

Since SDF-1 is highly expressed, lasting approximately 48 h after MI, approaches to inducing CXCR4 expression in MSCs cultured over the long term are clinically meaningful. To provide clinically effective homing, surface protein modification to incorporate recombinant CXCR4 protein on MSCs for several minutes has been developed [50]. VEGF-loaded layer-by-layer self-assembled encapsulated MSCs were able to release VEGF and SDF-1 continuously and result in MSC infiltration and inherent tropism to the MI zone, which was shown to be an effective and minimally invasive strategy for treating MI [51].

## Genetic manipulation

Because of the dissatisfactory quantity of SDF-1 expression after MI, HIF-1α transfection in the myocardium is a feasible method to induce SDF-1 expression. As a common regulatory gene, HIF-1 can induce the gene expression of VEGF and SDF-1α. MSC transplantation combined with HIF-1α transfection in the peri-infarcted region was shown to improve cardiac function 4 weeks after MI. Correspondingly, significant increases in VEGF and SDF-1α gene expression, angiogenesis, and MSC engraftment, as well as decreased cardiomyocyte apoptosis in peri-infarcted regions in the heart, were detected with adjuvant treatment [52].

Given that MSCs are minimally positive for CXCR4, transduction with a retroviral vector containing CXCR4 is an effective and efficacious method to enhance CXCR4 expression. MSCs modified with the CXCR4 gene have shown relatively increased migration towards SDF-1 but no negative effects on survival [53]. In another study, the upregulation of matrix metalloproteinases (MMPs) by CXCR4-overexpressing MSCs was thought to help cell engraftment in the collagenous tissue of the infarcted area. Adenoviral transduction of the CXCR4 gene into MSCs enhanced cell mobilization and engraftment into the ischaemic area and then promoted neomyoangiogenesis and cardiac repair after MI [54, 55]. The expression of VEGF and HIF-1 was also higher in MSCs overexpressing CXCR4, which enhanced neovascularization and endothelial differentiation after MI in in vivo research [56].

Treatment of MSCs transduced with adenoviral vector containing human SDF-1alpha was also accompanied by less infarct size and fibrosis, greater vascular density, better haemodynamic performance indexes, and thicker left-ventricular wall after transplantation [57]. Combined transfection of VEGF and SDF-1 in MSCs could have produced more VEGF and SDF-1 protein, which activated Akt expression and augmented the survival of the MSCs in vitro and in vivo. These results demonstrate that combination gene therapy including chemokines and angiogenic factors can enhance the therapeutic efficacy of MSC transplantation in terms of angiogenesis and cardiac repair after AMI [58].

Molecular studies have revealed that MSCs induce IGF-1 activation of survival proteins, including Akt and Bcl-xL, and inhibit the apoptosis protein glycogen synthase kinase 3beta; SDF-1 release from cells is also in parallel with IGF-1 expression. Massive mobilization and homing of stem cells have been observed in the infarcted myocardium due to increased SDF-1 expression after IGF-1-overexpressing MSC transplantation [59]. Transplantation of human MSCs overexpressing VEGF promotes SDF-1α expression in infarcted hearts and induces massive mobilization and homing of stem cells, which have also been related to the SDF-1/CXCR4 pathway in vitro and in vivo experiments [60]. Augmentation of PKCɛ expression has been shown to increase the therapeutic efficacy of MI in MSCs, including cell survival and differentiation, through the SDF-1/CXCR4 and PI3K/AKT pathways, which was demonstrated by an inhibitory experiment in which CXCR4 or PI3K partly attenuated the effects of PKCɛ-overexpressing MSCs [61]. Another gene has also been successfully modified in MSCs. Growth arrest-specific gene 6 (Gas6) encodes a protein that facilitates MSC treatment, including promoting cell chemotaxis, mitogenesis, and survival. The mechanism of Gas6-modified MSC therapy for postinfarcted heart failure involves enhanced HIF-1α-driven secretion of four major growth factors (VEGF, bFGF, SDF, and IGF-1) via enhanced Gas6/Axl autocrine prosurvival signalling and paracrine cytoprotective action [62]. Nestin, an intermediate filament protein, is regarded as a potential marker for MSCs. Nestin + BMSC transplantation had a higher chemotactic effect and repair efficacy in an AMI model by recruiting resident cardiac endothelial cells to the infarcted border region, which could also be accounted for by the SDF-1/CXCR4 pathway [63]. Nonviral transfection of the human SDF-1 gene into skeletal myoblasts was explored in an animal model, and transplanting these cells to establish transient tropism to favour extracardiac stem cell translocation to the infarcted zone was successful in optimizing chemotaxis [64].

## Hypoxic conditioning

Hypoxia increases the gene expression of HIF-1α and SDF-1. Knocking down the expression of HIF-1α reduces the expression of SDF-1 and terminates the migration of MSCs. The role of HIF-1α in hypoxia-induced MSC migration is mostly based on SDF-1-induced mobilization; therefore, hypoxia conditioning can be a reasonable strategy for the development of MSC-based therapeutics [65]. Preconditioning with the HIF-α prolyl hydroxylase inhibitor dimethyloxalylglycine has improved the survival capability and paracrine effects of MSCs with increased angiogenesis in vivo [66]. Hypoxic preconditioning of MSCs evokes an increase in the expression of large intergenic noncoding RNA-p21, HIF-1α, and CXCR4/7[67]. The optimal preparation conditions for MI before delivery under certain hypoxic environments have been investigated. MSCs cultured under normoxia rather than the cryopreserved state before 24-h hypoxia preconditioning emerge in a better state in terms of the expression of prosurvival, proangiogenic, and functional proteins that facilitate survival and engraftment in the infarct zone [68]. Serum deprivation and hypoxia can induce mitochondrial fragmentation with evidence of mitochondrial fission and apoptosis of MSCs, which can be inhibited by pretreatment with haemin, one of the heme oxygenase 1 activators [69].

MSCs with ageing show senescent characteristics compared with young counterparts. Preconditioning of aged MSCs with glucose depletion can enhance proliferation, delay senescence, and restore the ability of aged cells to repair senescent infarcted myocardium. Thus, preconditioning with glucose depletion is favourable to reduce the detrimental effect of ageing [70]. Under the same hypoxic conditions, MSCs derived from different sources showed distinct functional mechanism changes. Human placenta-derived MSCs exhibit greater chemotaxis with regard to SDF-1α-dependent cell migration and higher CXCR4 expression folds under CoCl_2_-induced hypoxia compared with human bone marrow-MSCs. The triggered mechanism pathways in human placenta-MSCs and human bone marrow-MSCs also differ under CoCl_2_-induced hypoxia [71]. Hypoxia in combination with laser treatments on human skeletal and cardiac muscle cells may recruit MSCs to migrate towards these treated cells; such a technique has therefore been applied as a potential technology for stem cell homing strategies [72].

## Ischaemic conditioning

Ischaemic preconditioning results in differential mobilization and recruitment of haematopoietic stem cells (HSCs) and MSCs in the early phase of cardioprotection. Ischaemic preconditioning in the early phase leads to a significant increase in circulating HSCs. In contrast, a rapid and prolonged decrease is accompanied by circulating MSC levels. The recruitment of HSCs and MSCs in the infarct and border zones is reversed by ischaemic preconditioning, and faster homing of MSCs than mobilization has also been observed [73].

In contrast to MSC priming on the CXCR4 receptor, the innovative research in our studies was devoted to determining how to modify the expression level of SDF-1 in the recipient. The systemic distribution of transplanted MSCs, regardless of their delivery route, was characteristic of lower engraftment in the heart but higher accumulation in the lungs. Although intramyocardial injection of MSCs into the ischaemic myocardium seemed to be more efficacious than other routes, such as intravenous and intracoronary delivery, there was still limited retention in the myocardium [74]. Ischaemic conditioning is a simple, safe, effective method to resist against ischaemic reperfusion injury through brief episodes of ischaemia. More importantly, remote tissue ischaemia can also induce the same cardioprotective efficacy, which sheds light on widespread clinical application. Conditioning is defined as pre-, per-, or post- if the timing of resorting to the therapeutics occurs prior to, during or after the index ischaemia accident, respectively [75]. Most likely is that remote ischaemic postconditioning is applied in clinical practice after the ischaemic event. We found that the SDF-1 protein concentration level in myocardial tissue was significantly improved after ischaemic infarction but only over a transitory period. Interestingly, remote ischaemic postconditioning could temporarily interrupt SDF-1 synthesis in the ischaemic myocardium [76]. Furthermore, synthesis could be restored after a lengthy interruption if remote ischaemic postconditioning was repeated periodically. Meanwhile, MSC retention in the myocardium was effectively enhanced after cell delivery. In contrast, the MSC distribution over the lungs and other peripheral solid organs was reversed [77]. Different operative access can give rise to different levels of inflammation and injury reactions, and minimally invasive access is associated with lower systemic inflammatory reactions compared with the traditional incision approach [78]. It has also been found that remote ischaemic conditioning can attenuate the inflammatory reaction in the ischaemic myocardium in rat models and in clinical trials [79, 80], which could potentially endanger the therapeutic efficacy and critical indexes in clinical practice.

## Device technique

During cardiac surgeries, manipulation of the procedure with cardioplegia and cardiopulmonary bypass results in an increase in circulating CXCR4-positive progenitor cells, which is also associated with increased myocardial SDF-1α expression in the atrial myocardium [81]. Transmyocardial revascularization (TMR) with a 10-channel needle which was used to improve the blood reperfusion from endocardial to epicardium in cardiac surgery performed in infarct rats subjected to left anterior descending coronary artery ligation has been shown to induce more MSC engraftment. The SDF-1 and CXCR4 levels were upregulated in TMR hearts within 1 week but dropped dramatically later [82].

Technologies harnessing ultrasound energy to deliver target genes into the myocardium have also been successfully employed in animal models. Multiple ultrasound-targeted delivery of stem cell factor and SDF-1 genes via microbubble destruction was shown to improve tissue perfusion and cardiac function after myocardial infarction [83]. Another study focused on determining the optimum ultrasound energy by vascular active factor expression, which was quantified by real-time quantitative reverse transcription polymerase chain reaction. The results showed that myocardial microenvironmental changes in terms of SDF-1 and VEGF in the 1.5 W/cm^2^ and 1 W/cm^2^ groups were markedly increased. Myocardial perfusion was markedly improved after synthetic therapy combined with MSC transplantation, based on the results of coronary angiography and 99 mTc-tetrofosmin scintigraphy [84]. Another active molecular NO microbubble combined with ultrasound technology also enhanced the therapeutic efficiency of MSCs in myocardial infarction by enhancing the SDF-1 and CXCR4 interaction [85].

Intramyocardial cell delivery has been shown to result in a higher level of angiogenic factor release. In contrast, intracoronary MSC delivery decreased CXCR4 expression with lower myocardial blood flow in a porcine MI model [86]. Meanwhile, subcutaneously implanted injectable hydrogels releasing SDF-1α in vivo presented more chemotaxis and resulted in a higher migratory ability of pretreated MSCs compared with systemic delivery and local injection [87]. Intramyocardial administration by transcatheter techniques (through a small-caliber needle lumen at different flow rates) did not remarkably disturb functionality, including clonogenicity, gene expression, and cytokine secretion, for cardiac tissue repair and regeneration [88]. Low-level shear stress can induce the secretion of SDF-1 and stimulate CXCR4 expression in human MSCs until the cells cover the wound zone of the MSC monolayer [89]. The SDF-1 expression level is associated with aetiological catalogues. SDF-1 is lower in ischaemic heart patients than in valvular heart patients and healthy populations, and beta-blocker treatment can significantly decrease SDF-1 levels [90]. Disease products, such as circulating HIV glycoprotein Gp120, can upregulate CXCR4 expression in MSCs [91].

## Conclusions

The prospect of MSC therapy in cardiovascular and inflammatory diseases might depend on the robustness of the pleiotropic functions of MSCs, which should be linked to their therapeutic efficacy. MSC transplantation remains a promising or competitive approach for treating myocardial ischaemia due to its promising preclinical efficacy and good safety profile. Despite its outstanding benefits in preclinical settings, the practical potency of MSCs remains controversial, since clinical trials with MSC application are feasible and largely safe but have not yet produced consistent benefits [92, 93]. Moreover, MSC recruitment into the heart induced by SDF-1 can possibly be followed by the transformation of these cells into fibroblasts rather than cardiomyocytes, which would exacerbate the pathophysiological conditions that worsen heart ejection function [94]. Consequently, an increasing number of studies have been conducted for a variety of clinical indications with novel paradigms rather than the classical concept of regeneration, suggesting that MSCs are still lagging in terms of their solid clinical translation. For this reason, our aim was to summarize priming methods available for still-promising cell types that, after more than half a century, have yet to reach their maturity [95]. Contextualizing these priming strategies with multiple dimension models emphasizes our ability to optimize this homing process and hinges on our understanding of its molecular characterization and biological mechanisms [96]. Most importantly, a deeper understanding of the innate mechanisms that govern the complex phenomenon of regeneration is essential to repairing or regenerating cardiac tissue [97]. Moving forward, it is only with a combined effort of basic medicine and translational work that the potential of MSC-based therapies can be realized [98].
